# Integrating Benzenesulfonic Acid Pretreatment and Bio-Based Lignin-Shielding Agent for Robust Enzymatic Conversion of Cellulose in Bamboo

**DOI:** 10.3390/polym12010191

**Published:** 2020-01-10

**Authors:** Xiaolin Luo, Zhenggang Gong, Jinghao Shi, Lihui Chen, Wenyuan Zhu, Yonghui Zhou, Liulian Huang, Jing Liu

**Affiliations:** 1 College of Materials Engineering, Fujian Agriculture and Forestry University, Fuzhou 350002, China; xluo@fafu.edu.cn (X.L.); LaoGongAY00@163.com (Z.G.); jinghao1884@163.com (J.S.); lihuichen@263.net (L.C.); 2 Jiangsu Provincial Key Laboratory of Pulp and Paper Science and Technology, Nanjing Forestry University, Nanjing 210037, China; ppzhuwy12@njfu.edu.cn; 3 Department of Civil and Environmental Engineering, Brunel University London, Kingston Lane, Uxbridge UB8 3PH, UK; Yonghui.Zhou@brunel.ac.uk

**Keywords:** benzenesulfonic acid pretreatment, components removal, lignin deposition, enzymatic hydrolysis, lignin-shielding agent

## Abstract

A hydrotrope-based pretreatment, benzenesulfonic acid (BA) pretreatment, was used to fractionate bamboo in this work. With optimized content (80 wt %) of BA in pretreatment liquor, about 90% of lignin and hemicellulose could be removed from bamboo under mild conditions (95 °C, 30 min or 80 °C, 60 min). The potential accessibility of BA pretreated substrate to cellulase was thus significantly improved and was also found to be much higher than those of acidic ethanol and dilute acid pretreatments. But the deposition of lignin on the surface of solid substrates, especially the BA pretreated substrate, was also observed, which showed a negative effect on the enzymatic hydrolysis efficiency. The addition of inexpensive soy protein, a bio-based lignin-shielding agent, could readily overcome this negative effect, leading the increase of enzymatic conversion of cellulose in BA pretreated substrate from 37% to 92% at a low cellulase loading of 4 FPU/g glucan. As compared to acidic ethanol and dilute acid pretreatments, the combination of BA pretreatment and soy protein could not only stably improve the efficiency of non-cellulose components removal, but also could significantly reduce the loading of cellulase.

## 1. Introduction

Adequate energy supply and healthy environment are the basic guarantees for the survival and development of human society. However, the endless energy and environmental issues caused by fossil fuels (such as gasoline and diesel) has posed a serious threat to global sustainable development [1]. Due to the advantages of high yield and renewable nature, transferring lignocellulose into biofuels (such as cellulosic ethanol) through biochemical methods has become one of the effective ways to solve the world’s most important energy and environmental problems [2]. 

Enzymatic hydrolysis and fermentation are the most commonly used methods for producing biofuels [3]. In order to overcome the natural recalcitrance of lignocellulose to enzymes, non-cellulosic components should be initially fractionated from lignocellulose by using pretreatments (especially chemical pretreatments) prior to enzymatic hydrolysis and fermentation [4]. In the past two decades, a large number of non-organic solvent-based chemical pretreatments (such as liquid hot water, dilute acid, alkali, sulfite and conventional organosolv pretreatments, etc.) have been developed and applied to different lignocelluloses [5,6]. However, in the process of improving the accessibility of cellulose in the pretreated substrate to enzyme, the energies consumed by these pretreatments are quite high (e.g., pretreatment temperature usually up to 150–220 °C) [7]. In order to improve the removal efficiency of non-cellulosic components with low energy consumption, some new solvents (e.g., deep eutectic solvents (DESs) [8] and hydrotropes [9]) derived pretreatments have been recently reported. For example, hydrotropes have hydrophobic and hydrophilic fractions in their molecular structures. Based on the amphiphilic structure of molecules, hydrotropes can improve the solubility of hydrophobic solutes in water through the aggregation effect [10]. For certain hydrophobic solute, different hydrotropes have different minimum hydrotropic concentration (MHC). Above the MHC, the hydrotropes can aggregate with the hydrotropic solute to form a water-soluble complex, resulting in higher aqueous solubility of hydrotropic solute [11]. Rencently, Zhu’s group reported a MHC of *p*-toluenesulfonic acid for lignin from poplar wood was 11.5 wt % in water [9]. Under same pretreatment temperature and time, they found that lignin removal was increased with the increase of *p*-toluenesulfonic acid concentration in water. Also, with a concentration of *p*-toluenesulfonic acid that ranged from 60–80 wt %, the pretreatment temperature and time were significantly decreased for achieving equivalent lignin removal as compared to conventional chemical pretreatments and/or traditional chemical pulping methods [9]. 

These new solvents-based pretreatments indeed significantly improved the potential accessibility of pretreated substrates at low pretreatment temperatures. However, during the processes of pretreatment cooling and solid substrate washing, part of the dissolved lignin may precipitate and then re-deposit on the inner and outer surfaces of the solid substrate. Due to significant non-productive adsorption, deposited lignin would result in a high level of enzyme loading to achieve decent cellulose enzymatic conversion [8,9]. Due to the high price of commercial enzymes (such as cellulase from Novozymes, etc.), a previous study [12] found that the cost of commercial enzyme used in enzymatic hydrolysis process has accounted for at least 30–50% of the overall biofuel production costs. Therefore, the high cost of enzymes induced by non-productive adsorption would limit the further development of new solvent derived pretreatments, even with high non-cellulosic components removal. 

At present, the addition of lignin-shielding agent to the enzymatic buffer solution is regarded as an effective method for overcoming the non-productive adsorption of deposited lignin to cellulase during the enzymatic hydrolysis process [13]. In general, the lignin-shielding agent can be divided into the following three categories: (i) non-catalytic proteins (such as bovine serum albumin [14] and soy protein [15,16], etc.); (ii) surfactants (such as polyethylene glycol (PEG) [17] and polyvinylpyrrolidone (PVP) [18], etc.); (iii) metal ions (such as Ca^2+^ and Mg^2+^, etc.) [19]. Although non-catalytic proteins are biocompatible and efficient, commercial bovine serum albumin is relatively expensive for industry application [12]. Surfactants and metal ions are relatively inexpensive, but these additives adversely affect the activity of fermenting microorganisms and complicate the post-treatment process of the fermentation broth [20]. 

Through examining these issues faced by current biofuels industry, we think that efforts regarding two aspects are still needed to make the biofuel production process more economical. The two aspects include (1) producing substrates with high potential accessibility, and (2) reducing the enzyme loading by developing an inexpensive bio-based lignin-shielding agent. Herein, we firstly reported the integration of benzenesulfonic acid (BA) pretreatment and inexpensive soy protein for robust enzymatic conversion of cellulose in bamboo. BA pretreatment could obtain the substrate with high potential accessibility through efficient removal of non-cellulosic components from bamboo at low temperature. Based on our previous research, soy protein isolated from inexpensive defatted soy flour by enzymatic hydrolysis buffer solution could also effectively block the lignin deposits formed during the process of BA pretreatment, resulting in decent cellulose enzymatic conversion at low enzyme loading. We expect that the integration of benzenesulfonic acid (BA) pretreatment and inexpensive soy protein can not only improve the potential accessibility of cellulose in pretreated solid substrate to enzymes, but can also reduce the enzyme loading for producing fermentable sugar from lignocellulose. 

## 2. Materials and Methods

### 2.1. Materials

Bamboo particles (40–80 mesh) and defatted soy flour were generously provided by Fujian Qingshan Paper Industry Co., Ltd. (Sanming City, China) and Beijing Yaoyou Technology Co., Ltd. (Beijing City, China), respectively. Benzenesulfonic acid (BA) and ethanol with an analytical grade were purchased from Aladdin^®^ Reagent Co., Ltd. (Shanghai City, China). We purchased commercial enzymes (cellulase, Celluclast 1.5 L^®^; cellobiase, Novozyme 188), monomer sugar standards (arabinose, glucose and xylose, etc.), bovine serum albumin, and microcrystalline cellulose (Avicel) from Sigma-Aldrich Co., Ltd. (Shanghai City, China) and used them as received. We also ordered Pierce™ BCA Protein Assay Kit from Thermo-Fisher Scientific life (Rockford, IL, USA).

### 2.2. Pretreatments

Prior to enzymatic hydrolysis, bamboo particles were pretreated by using BA, acidic ethanol (ET), and dilute acid (DA) pretreatments, respectively. All pretreatments were conducted in a round bottom flask with a condenser. The mass ratio of the pretreatment liquor to the bamboo particles (on an o.d. basis) for all pretreatments was fitted as 15:1 (*g*:*g*). After pretreatment, the whole pretreatment slurry was separated by vacuum filtration on a Büchner funnel. Resultant solid substrates were then washed by deionized water until the pH of filtrate reached 6.5–7. Based on the moisture content, pretreatment induced solid substrate yield (SSY) could be gravimetrically measured.

For BA pretreatments, BA was initially dissolved in deionized water and resultant mixture was used as the pretreatment liquor. The mass content of BA in the pretreatment liquor, pretreatment temperature, and time of BA pretreatments ranged from 0–90 wt%, 50–110 °C, and 30–60 min, respectively. 

For ET pretreatments, two levels of ethanol (ET) and sulfuric acid were used. First, 23.6 g of ET (0.51 mol) and 25.1 g of concentrated sulfuric acid (0.26 mol) were mixed with deionized water to configure a pretreatment liquor. The molar quantities of ET and H^+^ released from sulfuric acid were assumed to be equivalent to the mole of 60 wt % BA in pretreatment liquor. The corresponding pretreatment was abbreviated as ET(1) to simplify following discussion. Second, 31.5 g of ET (0.68 mol, equivalent to the mole of 80 wt % BA in pretreatment liquor) and 33.5 g of sulfuric acid (0.34 mol) were loaded into another pretreatment liquor, for which the pretreatment was termed as ET(2) pretreatment. The ET(1) and ET(2) pretreatments were conducted at 90 °C for 30 min and 80 °C for 60 min, respectively.

For DA pretreatments, the loadings of sulfuric acid, pretreatment temperature, and time were the same as those of the above two ET pretreatments. Resultant pretreatments were also abbreviated as DA(1) and DA(2), respectively. 

### 2.3. Solid Substrates Composition and Pretreatment Liquor Analysis

Bamboo raw material (RM) and washed solid substrates were firstly vacuum-dried at 35 °C overnight and then hydrolyzed by using a well-known two-steps sulfuric acid hydrolysis process. Overall, the contents of carbohydrates (cellulose and hemicellulose) and Klason lignin in samples were measured according to the method reported by the US National Renewable Energy Laboratory [21]. This method was developed based on an ASTM E1758-01 standard method titled “Standard method for the Determination of Carbohydrates by HPLC” [21]. Based on the measured content, the component (cellulose, hemicellulose, or Klason lignin) removal (%) induced by pretreatment was thus calculated as:

(1) $$\mathrm{Component}\;\mathrm{removal}=\;100\frac{\left[C_{1}-\;\left(\mathrm{SSY}/100\right)\;\times {\;C}_{2}\right]}{C_{1}}$$

where SSY is the solid substrate yield (%, *w*/*w*, on o.d. basis of raw material), while C_1_ and C_2_ are the contents of components in raw material (%, *w*/*w*) and pretreated solid substrate (%, *w*/*w*, on o.d. basis of substrate), respectively.

Monomer sugars in pretreatment liquor were measured using a Dionex IC system (ICS 5000^+^, ThermoFisher, Waltham, MA, USA) equipped with an integrated amperometric detector and Carbopac™ PA20 analytical columns at 25 °C [21]. The concentration of furfural in pretreatment liquor was also analyzed by the Dionex ICS 5000^+^ IC system equipped with a Supelcogel C-610H column at temperature 30 °C and a UV detector at 210 nm [9].

### 2.4. Lignin Separation from BA-W Pretreatment Hydrolysate

Lignin, termed as BA80 lignin, was separated from pretreatment hydrolysate that resulted from the BA pretreatment with 80 wt % BA in pretreatment liquor at 80 °C for 60 min. The pretreatment hydrolysate was initially diluted 10 times with deionized water and then centrifuged at 8000 rpm for 10 min. After removing the supernatant, the obtained lignin was dialyzed in a dialysis bag with a molecular weight cut off of 1000 Da until the pH of the dialysate reached neutral. Resulting lignin-water mixture in the dialysis bag was further divided into two portions. One portion was filtrated and freeze-dried to obtain dried lignin, which was used for subsequent enzymatic hydrolysis of Avicel. Another portion was directly diluted by acetic acid-sodium acetate (AA-SA) buffer solution (pH 5.0 and 50 mmol/L) for dynamic light scattering (DLS) measurement. 

### 2.5. Enzymatic Hydrolysis

With a solid loading of 2% (*w*/*v*), pretreated solid substrates and Avicel were enzymatically hydrolyzed in AA-SA buffer solution (pH 5.0, 50 mmol/L) at 50 °C and 150 rpm for 72 h. To overcome the negative effect of deposited lignin on the enzymatic hydrolysis, we extracted the water-soluble soy protein from defatted soy flour using an AA-SA buffer solution (pH 5.0, 50 mmol/L) and ultrasonic treatment, as described previously [15]. After removing the defatted soy flour residue by centrifugation (5000 rpm, 5 min), the supernatant containing the water-soluble soy protein was used directly for the enzymatic hydrolysis experiment. The concentration of water-soluble soy protein in supernatant was determined through a bicinchoninic acid (BCA) method using the Pierce™ BCA Protein Assay Kit as a chromogenic agent. To test the lignin shielding effect of soy protein, specific volume, or mass of soy protein supernatant, AA-SA buffer solution, and substrate were mixed at 50 °C and 150 rpm for 2 h prior to enzyme addition. The volume of soy protein supernatant used for enzymatic hydrolysis was calculated based on soy protein loading (mg/g substrate) and its protein concentration (mg/mL). In contrast, the enzyme was added directly to the enzymatic hydrolysis medium. The starting time of enzyme hydrolysis was fixed as the time of adding enzyme for all hydrolysis experiments. The loading of cellulase and soy protein ranged from 1–10 FPU/g glucan and 20–320 mg soy protein/g substrate, respectively. Cellobiase loading (CBU/g glucan) was 1.5 times of cellulase (FPU/g glucan) for each hydrolysis experiment. After enzymatic hydrolysis, we used a sugar analyzer (2900D, YSI Inc., Yellow Springs, OH, USA) to determine the concentration of glucose in the enzymatic hydrolysate. Based on glucose concentration, enzymatic conversion (%) of cellulose in pretreated substrate and corresponding glucose yield (%) could be calculated as follows:

(2) $$\mathrm{Enzymatic}\;\mathrm{conversion}=\;100\frac{C_{\mathrm{glu}}\;\times \;V\;\times 0.9}{M_{\mathrm{Cell}}}$$

(3) $$\mathrm{Glucose}\;\mathrm{yield}=\;\mathrm{Enzymatic}\;\mathrm{conversion}\times \left(1-\frac{R_{\mathrm{cell}}}{100}\right)$$

where C_glu_, V, M_cell_, and R_cell_ are the concentration of glucose in the enzymatic hydrolysate (g/L), the volume (L) of enzymatic hydrolysate, the mass of the cellulose in the substrate used for enzymatic hydrolysis, and the removal (%) of cellulose in substrate pretreated by the pretreatment, respectively. 

### 2.6. Characterization of Raw Material and Solid Substrates

The surfaces of vacuum-dried bamboo raw material and pretreated substrates were firstly coated by the gold via vacuum sputtering and then imaged by a field emission scanning electron microscopy (FE-SEM, SU8220, Hitachi, Tokyo, Japan) under a high vacuum operation mode at 3.0 kV. 

After degassing, we used a physical adsorption instrument (Version 5.0, Quantachrome Instruments, Boynton Beach, FL, USA) to determine the total pore volume and BET surface area of the vacuum-dried bamboo raw material and pretreated substrates. Nitrogen was used as the probe gas during the adsorption and desorption processes. The temperature of the entire measurement process was maintained at 300 K.

According to the method reported in the aforementioned literature [22], we used X-ray photoelectron spectroscopy (XPS, ESCALAB250, Thermo-Fisher Scientific, Waltham, MA, USA) equipped with a monochromatic Al-Kα X-ray source to determine the relative content of C and O elements on the surface of the samples (bamboo raw material and pretreated substrates). The XPS survey spectra were collected from 200 to 600 eV. Surface lignin coverage (S_lig_, %) of the sample was further calculated based on the empirical equation developed by Laine et al. [22]. This empirical equation can be expressed as:

(4) $$S_{\mathrm{lig}}=\;100\frac{O/C_{\left(\mathrm{Sample}\right)}\;-\;O/C_{\left(\mathrm{Carbohydrate}\right)}}{O/C_{\left(\mathrm{Lignin}\right)}\;-\;O/C_{\left(\mathrm{Carbohydrate}\right)}}$$

where O/C_(Sample)_, O/C_(Carbohydrate)_ and O/C_(Lignin)_ are the ratios of O to C element on the surface of the sample, bleached kraft pulp (0.83, reported by reference [22]) and milled wood lignin (0.33, reported by reference [22]), respectively.

### 2.7. DLS Analysis

Cellulase, soy protein (Section 2.5), and BA80 lignin (Section 2.4) samples were initially diluted by AA-SA buffer solution (pH 5.0 and 50 mmol/L) to the concentrations of 0.1, 0.1, and 0.01 mg/mL, respectively. Diluted cellulase and soy protein solutions were further filtered by using 0.22 μm Nylon membranes to remove some insoluble particles. Then, we used a DLS analyzer (Zetasizer NanoZS90, Malvern Instruments, Malvern, UK) to measure the zeta potential and particle size of all samples at 25 °C. For the DLS size measurements of cellulase-BA80 lignin and soy protein-BA80 lignin mixtures, the volume ratio of enzyme or soy protein to lignin was set at 4:1 (*v*/*v*).

## 3. Results and Discussion

### 3.1. Effects of Different Pretreatments on the components Removal and Cellulose Enzymatic Conversion

In order to examine the performance of BA pretreatment, ET and DA pretreatments were selected as the controls. The effects of these three pretreatments on the components removal and cellulose enzymatic conversion were preliminarily evaluated. 

**Figure 1:**
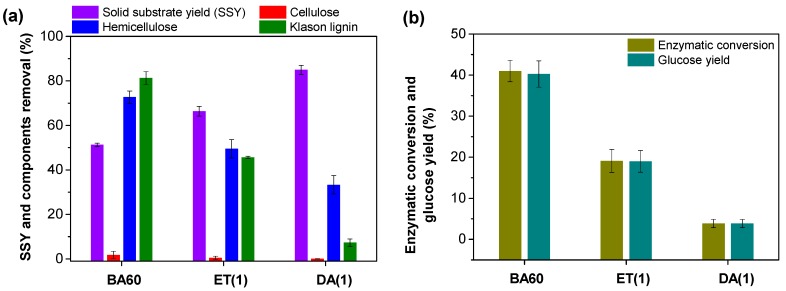
Effects of different pretreatments (BA60, ET(1), and DA(1)) on the (**a**) components removal and (**b**) enzymatic hydrolysis efficiency. Experiments including pretreatment, enzymatic hydrolysis, and corresponding measurements were repeated twice. The measurement errors were the standard deviations of the replicate analyses. To simplify the discussion, the BA pretreatment conducted with a content of 60 wt % BA in pretreatment liquor at 90 °C for 30 min was abbreviated as BA60 pretreatment. Under same pretreatment temperature and time, the ET and DA pretreatments were also abbreviated as ET(1) and DA(1), respectively, while the molar amounts (0.51 mol) of ethanol and hydrogen ions were both equivalent to that of BA used in BA60 pretreatment.

Under the identical conditions, BA60 pretreatment obtained the lowest solid substrate yield (SSY, 51.24%, Figure 1a) and the highest cellulose content (74.78%, Table S1) as compared with ET(1) and DA(1) pretreatments, indicating a high selectivity of the BA60 pretreatment for removing non-cellulosic components (lignin and hemicellulose) from bamboo. As shown in Figure 1a, the lignin and hemicellulose removal of BA60 pretreatment reached 81.3% and 72.7%, which were much higher than those of ET(1) (45.6% and 49.5%) and DA(1) (7.3% and 33.3%) pretreatments. This may be due to the hydrotrope nature of aromatic sulfonic acids (e.g., *p*-toluenesulfonic acid (*p*-TsOH) and BA, etc.). When the concentration of the added hydrotropes is above its specific minimal hydrotrope concentration (MHC), these hydrotropes can increase the solubility of hydrophobic substances (such as lignin) through aggregation [9]. Because of high non-cellulosic component removal, with a cellulase loading of 10 FPU/g glucan, the cellulose enzymatic conversion and glucose yield of the substrate pretreated by BA60 pretreatment were 2 and 10 times higher than those of ET(1) and DA(1) pretreatments, respectively. Unlike the expected outcomes, the relatively high non-cellulosic components removal of BA60 pretreatment did not result in satisfactory accessibility or digestibility of cellulose in the resultant solid substrate. At relatively high cellulase loading (10 FPU/g glucan), corresponding cellulose enzymatic conversion was still below 50% (Figure 1b), which was much lower than those of other new solvents (e.g., DESs and *p*-TsOH with enzymatic conversion beyond 70%) derived pretreatments conducted at optimized conditions [9,10]. Therefore, the conditions of BA pretreatment need to be further optimized.

### 3.2. Preliminary Optimization of BA-W Pretreatment

**Figure 2:**
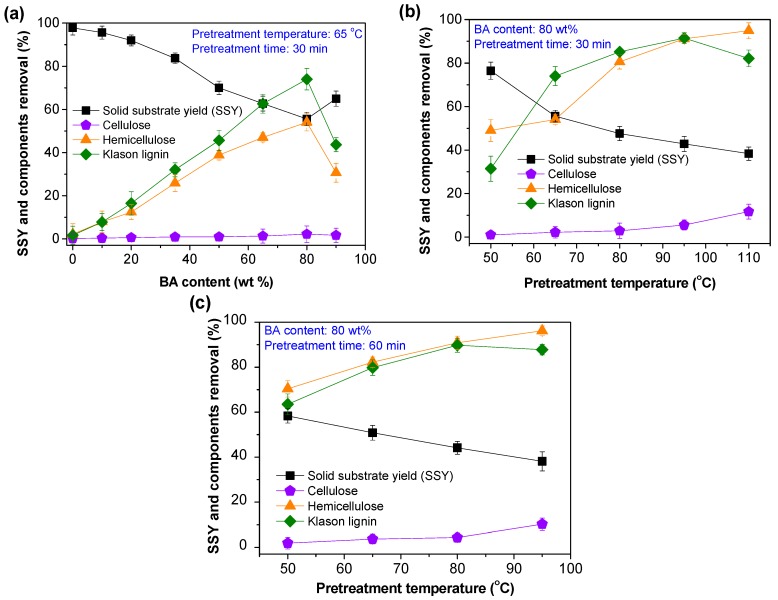
Effects of the pretreatment conditions on the solid substrate yield (SSY) and components removal. (**a**) BA content effects at 65 °C for 30 min; (**b**,**c**) temperature effects at a BA content of 80 wt % for (**b**) 30 min and (**c**) 60 min, respectively.

**Figure 3:**
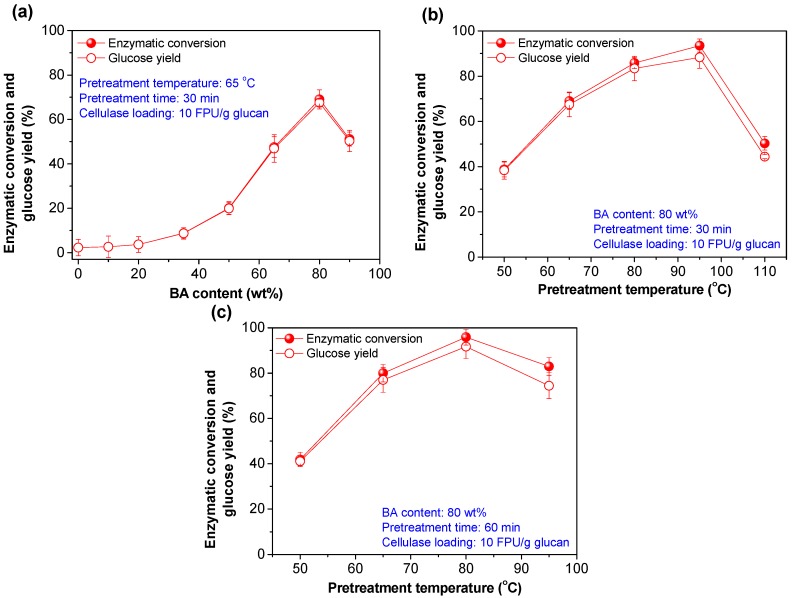
Effects of the pretreatment conditions on the enzymatic hydrolysis efficiency. (**a**) BA content effects at 65 °C for 30 min; (**b**,**c**) temperature effects at a BA content of 80 wt % for (**b**) 30 min and (**c**) 60 min, respectively.

To examine the BA content effect, BA pretreatments were conducted at a temperature of 65 °C and reaction time of 30 min. Overall, when the content of BA in pretreatment liquor was increased from 0 to 80 wt %, lignin and hemicellulose removal gradually increased from 1.7% and 2.2% to 74.0% and 54.0% (Figure 2a), respectively. This result may be explained as suggesting that the increase of aromatic hydrotrope acid content can improve the acidity and hydrotropic efficiency of the pretreatment liquor, which is beneficial to the acid hydrolysis of non-cellulose components and the dissolution of lignin via aggregation [9,23,24]. During this process, cellulose removal was found to be lower than 3%, possibly due to high crystallization properties. Further increasing the BA content to 90 wt % resulted in the decreases of lignin and hemicellulose removal from the highest values to 43.7% and 30.7% (Figure 2a), respectively. This phenomenon may be ascribed to the high viscosity of the hydrotrope-water system [25], which inevitably influenced the mass transfer efficiency of solvent and dissolved components during BA pretreatment. Accordingly, the maximum cellulose enzymatic conversion and glucose yield were also achieved at the optimized BA content of 80 wt % (Figure 3a).

With an optimized BA content of 80 wt %, the pretreatment temperature effect was investigated with time variations. When pretreatment time was fixed as 30 min, up to 94.9% of hemicellulose and 11.7% cellulose in the bamboo raw material were successively removed with the increasing of the pretreatment temperature (Figure 2b). However, a slight decrease of lignin removal was observed from the maximum value (91.4%) at 95 °C to 82.1% at 110 °C (Figure 2b). The different dissolution behaviors of carbohydrates and lignin can be mainly attributed to two reasons. First, high pretreatment temperatures can accelerate acid-catalyzed hydrolysis of carbohydrates [26]. Second, under low-temperature conditions (e.g., temperature ≤ 95 °C), the dominant reactions are the cleavages of β-aryl ether bonds and aggregation [9], which are beneficial for lignin degradation and dissolution. But at high-temperature conditions (e.g., temperature > 95 °C), the dominant reaction may shift to the lignin condensation. Previous research [27,28,29] demonstrated that condensed lignin was preferable to deposit on the surface of solid substrate during acid catalyzed pretreatments. 

In summary, BA pretreatments at high temperature may not only cause the lignin condensation but also the deposition of condensed lignin. In addition to carbohydrates removal, the potential accessibility of cellulose in pretreated substrates was very likely the balance of these two parallel reactions; lignin degradation and deposition. Notably, with a cellulase loading of 10 FPU/g glucan, the highest cellulose enzymatic conversion was also obtained at a pretreatment temperature of 95 °C for 30 min (Figure 3b). 

For the BA pretreatment series with a pretreatment time and BA content of 60 min and 80 wt %, the effects of the pretreatment temperatures on the components removal (Figure 2c) and cellulose enzymatic conversion (Figure 3c) were similar to those of pretreatments conducted with a short pretreatment time (Figure 2b and Figure 3b). Finally, the BA content, pretreatment temperature, and time were preliminarily optimized as 80 wt %, 95 °C, and 30 min, or 80 wt %, 80 °C, and 60 min.

### 3.3. Components Removal and Enzymatic Conversion of Substrates Pretreated at Optimized Conditions

**Figure 4:**
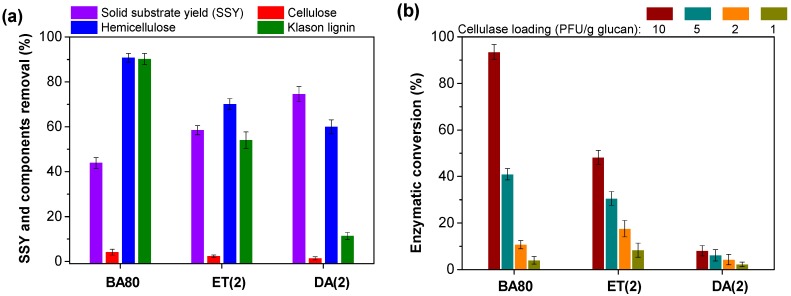
Effects of different pretreatments (DA(2), ET(2), and BA80 pretreatments) conducted at optimized conditions on the (**a**) components removal and (**b**) enzymatic hydrolysis efficiency. All pretreatments were conducted at 80 wt % BA content and 80 °C for 60 min.

Similar to the results shown in Figure 1, lignin and hemicellulose removal (90.2% and 90.8%, Figure 4a) of substrate pretreated by BA pretreatment at 80 wt % BA (0.68 mol) and 80 °C for 60 min (abbreviated as BA80 pretreatment) were also much higher than those of ET and DA pretreatments (abbreviated as ET(2) and DA(2)) with equimolar loadings of ethanol and hydrogen ions. 

The effects of non-cellulosic components removal on the potential accessibilities of cellulose in these substrates were thereby evaluated by conducting enzymatic hydrolysis with different cellulase loading (1, 2, 5, and 10 FPU/g glucan). It can be seen that with the decrease of cellulase loading from 10 to 1 FPU/g glucan, the cellulose enzymatic conversion reductions of 37%, 64%, and 83% for ET(2) and 24%, 47%, and 72% for DA(2) derived substrates were significantly lower than that (56%, 89%, and 96%) of substrate from BA80 pretreatment (Figure 4b). But the enzymatic conversions of cellulose in ET(2) and DA(2) substrates at high cellulase loading of 10 FPU/g glucan were only 48% and 8%. These results may be associated with two factors: (1) potential substrate accessibility-limited cellulose enzymatic conversion; (2) surface lignin deposits-limited cellulose enzymatic conversion. During the enzymatic hydrolysis process, part of the enzymes adsorbs irreversibly with lignin deposited on the surface of the substrate [30]. More enzymes will disperse freely in enzymatic hydrolysis buffer solution if the substrate possesses less lignin deposits. As a result, the substrate with low non-cellulosic components removal will be highly recalcitrant to the enzymes, resulting in poor cellulose digestibility within the 72-h hydrolysis process [15,31]. Conversely, for the substrate with high potential accessibility, the digestion of cellulose can be completely preceded by the residual enzymes during the 72-h hydrolysis process, although many more enzymes have been adsorbed by the substrate with more lignin deposits [32]. For example, near-complete conversion (93.5%, Figure 4b) of cellulose in BA80 pretreated substrate was successfully achieved by using the 72-h enzymatic hydrolysis with a cellulase loading of 10 FPU/g glucan. Overall, regardless of the amount of enzyme, the high accessibility of substrate is the prerequisite for robust cellulose enzymatic conversion.

### 3.4. Characterizations of the Substrates Pretreated at Optimized Conditions

**Figure 5:**
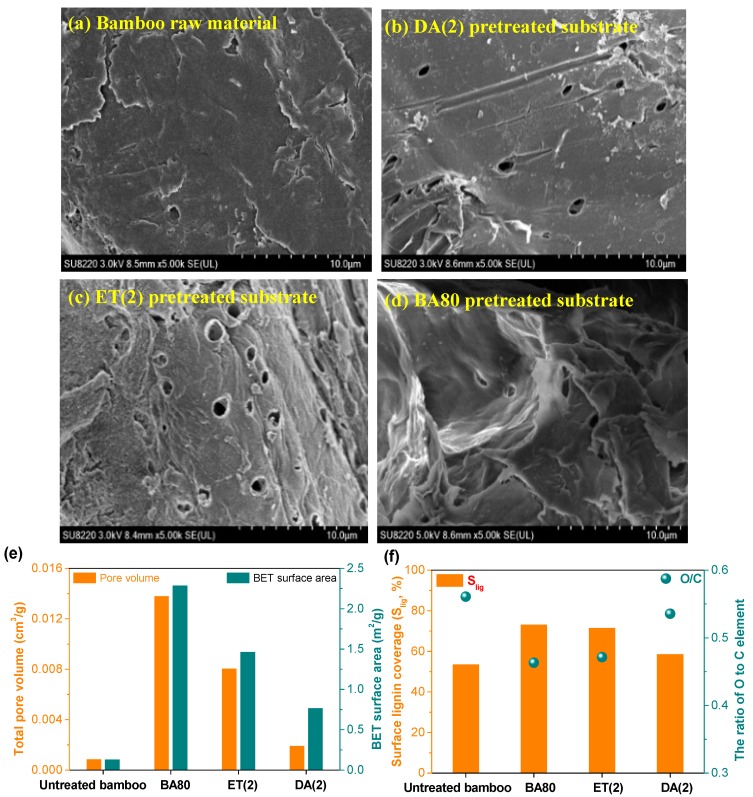
(**a**–**d**) SEM images, (**e**) total pore volume and BET surface area, and (**f**) surface lignin coverage (S_lig_) of bamboo raw material and pretreated solid substrates. Three pretreatments (DA(2), ET(2), and BA80) were conducted under optimized conditions that are detailed in Figure 4.

To verify the effect of potential accessibility on the enzymatic hydrolysis efficiency, bamboo raw material and the solid substrates pretreated by the three pretreatments were characterized here. Previous studies [4,5,6,7] have found that the cell walls of woody biomass will be gradually destroyed by the removal of non-cellulosic components, particularly lignin. After three pretreatments, some circular or irregular pores (Figure 5b–d) were observed on the surface of the pretreated solid substrates. Since most of the lignin and hemicellulose were removed (>90%, Figure 4a) during the BA80 pretreatment, the morphology of the pretreated substrate (Figure 5d) was completely deconstructed, resulting in larger pores (Figure S1d) than those of ET(2) and DA(2) pretreatments (Figure S1b,c). Correspondingly, the total pore volume and BET surface area of the BA80 pretreated solid substrate reached 0.0138 cm^3^/g and 2.29 m^2^/g (Figure 5e), respectively, which were 1.7, 7.1, 15.7, and 1.6, 3.0, and 16.8 times of ET(2) and DA(2) derived substrates and bamboo raw material. For the pores with the diameters larger than the average diameter of the cellulase molecule (5.1 nm) [33], the cumulative pore volume (0.0137 cm^3^/g, Figure S2 and Table S2) of BA80 pretreated substrate was also significantly higher than those of other two pretreatments. These results explain the above hypothesis that at higher enzyme loading, the enzymatic hydrolysis efficiency is primarily determined by the accessibility of cellulose in the pretreated solid substrate to the enzyme. 

However, based on XPS spectra (Figure S3), the ratio of element O to C on the surface of BA80 pretreated solid substrate was also lower than those of other two pretreatments, resulting in higher lignin surface coverage (S_lig_, 73.4%, Figure 5f). This may also be responsible for the rapid decrease of enzymatic conversion of cellulose in the BA80 pretreated solid substrate with the reduction of cellulase loading (Figure 4b). According to previous reports [34,35] and above characterization results, deposited lignin particles may not significantly affect the potential accessibility (pore volume or surface area) of the pretreated substrate, but they will influence the actual accessibility of cellulose in substrate to enzymes. This is mainly because of that the lignin deposits can significantly impede direct contact or adsorption between cellulose and enzyme molecules through non-productive adsorption [36]. It is therefore foreseeable that with high potential accessibility, the cellulose enzymatic conversion can be expected to be significantly increased at lower enzyme loading, if the deposited lignin on the surface of the BA80 pretreated solid substrate is effectively shielded or blocked. 

### 3.5. Improving the Enzymatic Conversion of Cellulose in Pretreated Substrates by Inexpensive Soy Protein

Although high cellulose enzymatic conversion was achieved by BA pretreatments conducted at optimized conditions, the enzyme loading used for enzymatic hydrolysis process was still as high as 10 FPU/g glucan. In order to reduce the enzyme loading, a readily available and abundant protein—soy protein, which could be simply separated from inexpensive defatted soybean flour based on our previous report [15], was used to shield the lignin deposited on the surface of the different pretreated substrates. 

**Figure 6:**
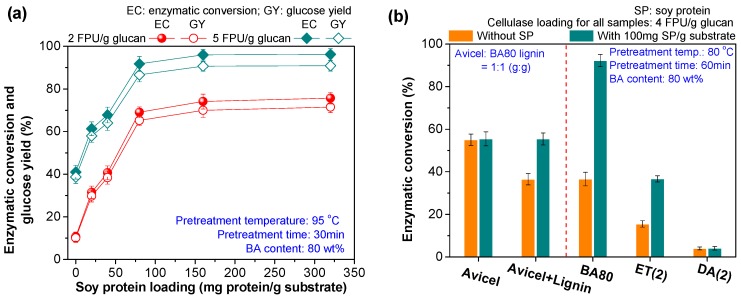
(**a**) Effect of soy protein loading on the enzymatic conversion of cellulose in BA80 pretreated substrate at two enzyme loading (2 and 5 FPU/g glucan); (**b**) comparisons of promotion effects of soy protein on Avicel and substrates from different pretreatments.

For the substrate pretreated by BA pretreatment at optimized conditions (80 wt % BA content, 95 °C and 30 min), cellulose enzymatic conversion significantly increased (Figure 6a) with the addition of soy protein at two enzyme loading (2 and 5 FPU/g glucan). When the soy protein loading reached a 100 mg protein/g substrate, resultant cellulose enzymatic conversions basically reached equilibrium values. Further increasing the soy protein loading did not result in significant improvement of the cellulose enzymatic conversion within 72-h enzymatic hydrolysis (Figure 6a), possibly due to the lignin deposits being sufficiently blocked at a soy protein loading of 100 protein/g substrate. A similar promoting effect (Figure 6b) was also observed on another BA pretreated substrate (80 wt % BA, 80 °C and 60 min), indicating that soy protein could effectively overcome the negative effect of deposited lignin on the cellulase. With the addition of soy protein, the enzymatic conversion of cellulose in BA80 pretreated substrate could increase from 37% to 92% at a low cellulase loading of 4 FPU/g glucan (Figure 6b). In other words, soy protein addition could reduce the cellulase loading by 2.5 times (10 to 4 FPU/g glucan) for achieving the equivalent level of cellulose enzymatic conversion (−93%, Figure 4b). However, with the same cellulase loading (4 FPU/g glucan), the effect of soy protein on the enzymatic conversions of cellulose in the solid substrates pretreated with ET(2) and DA(2) pretreatments was obviously lower than that of BA80 pretreatment (Figure 6b). 

**Figure 7:**
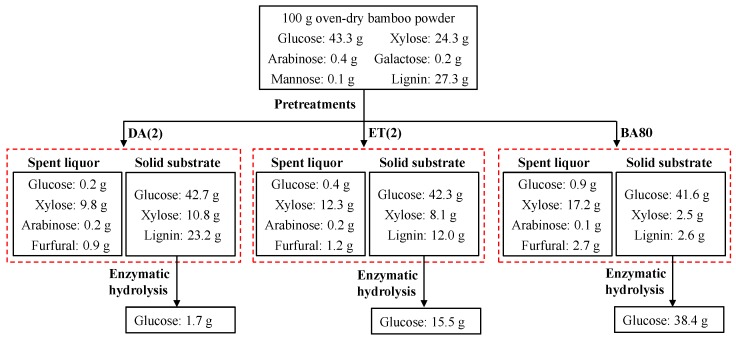
Mass balance of glucose during DA(2), ET(2), and BA80 pretreatments and enzymatic hydrolysis. Three pretreatments (DA(2), ET(2), and BA80) were conducted under optimized conditions that are detailed in Figure 4. Cellulase and soy protein loadings of the enzymatic hydrolysis were 4 FPU/g glucan and 100 mg protein/g substrate.

To compare the performance of DA, ET, and BA pretreatments for recovering fermentable glucose, a mass balance of glucose during DA(2), ET(2), and BA80 pretreatments and enzymatic hydrolysis was conducted (Figure 7). From 43.3 g glucose in 100 g oven-dry bamboo powder, measured glucose in spent liquor and pretreated solid substrate were 0.2 and 42.7 g for DA(2), 0.4 and 42.3 g for ET(2), and 0.9 and 41.6 g for BA80 pretreatments, respectively. The recoveries of glucose for these three pretreatments were similar during the pretreatment process. However, the recovery of glucose (88.7%) from BA80 pretreated substrate during enzymatic hydrolysis was much higher than those of DA(2) (3.9%) and ET(2) (35.8%) pretreatments. This is probably due to the integration of the high potential accessibility of cellulose in BA80 pretreated substrate and shielding effect of soy protein to lignin deposited on the surface of pretreated substrate. These results clearly indicated that under same pretreatment severity and enzymatic hydrolysis conditions, BA pretreatment is superior to DA and ET pretreatments for the recovery of glucose from bamboo. 

These results confirm our preceding hypothesis that the enzymatic hydrolysis efficiency of cellulose in the substrate with low non-cellulosic components removal is mainly limited by its potential accessibility [15,32], while the enzymatic hydrolysis efficiency of substrate with high potential accessibility is mainly affected by the surface deposition of lignin [26,34,36,37,38,39,40]. It is thereby noteworthy that the integrating BA pretreatment and inexpensive soy protein will be a reliable way for improving the non-cellulosic components removal efficiency at low temperature and achieving decent cellulose enzymatic conversion at low enzyme loading. 

Other than pretreated solid substrate, the effect of soy protein on the enzymatic hydrolysis efficiency of pure cellulose (Avicel) was also investigated. It was found that the addition of soy protein resulted in no changes in the enzymatic conversions of Avicel. However, in the absence of soy protein, the cellulose enzymatic conversion of the Avicel-lignin mixture (1:1, *g*:*g*) decreased from 55.4% to 36.5% (Figure 6b), while the corresponding enzymatic conversion of this mixture was recovered to initial level with the addition of 100 mg soy protein/g mixture. These results demonstrate two facts: (1) soy protein has no effect on the enzyme and does not interact with cellulose; (2) the promotion effect of soy protein mainly relies on shielding the lignin deposited on the surface of solid substrates. This is consistent with the previously reported mechanism of bovine serum protein for promoting enzymatic hydrolysis efficiency [38]. Bovine serum albumin mainly adsorbs to the surface of lignin rather than cellulose through hydrophobic interaction.

**Figure 8:**
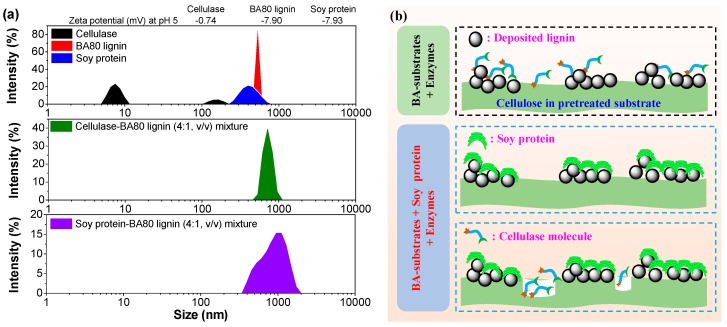
(**a**) Zeta potential and particle size of cellulase, BA80 lignin, soy protein and their mixtures; (**b**) a schematic diagram for promoting the enzymatic conversion of cellulose in BA80 pretreated solid substrate by using soy protein.

In the AA-SA buffer at a pH value of 5, it was found that the surfaces of lignin isolated from the BA80 pretreatment hydrolysate, and soy protein were both negatively charged (zeta potential of −7.90 and −7.93, Figure 8a), indicating that these two substances are not likely to be adsorbed through electrostatic interaction. However, when cellulase and soy protein were mixed separately with BA80 lignin in same buffer solution, it was further found that the particle size for either cellulase-lignin or soy protein-lignin colloid was larger than that of cellulase, lignin, or SP itself (Figure 8a). Therefore, based on the above experimental results, a schematic diagram (Figure 8b) for promoting the enzymatic conversion of cellulose in BA80 pretreated solid substrate by soy protein was finally proposed as: (1) soy protein may adsorb to the surface of lignin through hydrophobic interaction; (2) soy protein-lignin complex has no interaction with cellulase; (3) more free enzymes can be used to hydrolyze cellulose in the solid substrate as compared to that without a soy protein addition.

## 4. Conclusions

In this work, a hydrotrope (benzenesulfonic acid, BA)-based pretreatment was applied to fractionate bamboo at low temperatures. The efficiency of BA pretreatment for removing non-cellulosic components from bamboo was significantly higher than those of acidic ethanol (ET) and dilute acid (DA) pretreatments. As a result, BA pretreatment achieved relatively high potential accessibility of cellulose in substrates to enzymes, resulting in satisfactory cellulose enzymatic conversion at a cellulase loading of 10 FPU/g glucan. By using inexpensive soy protein to block the lignin deposited on the surface of BA80 pretreated substrate, equivalent cellulose enzymatic conversion could be obtained at a low cellulase loading (4 FPU/g glucan). Thus, the integration of BA pretreatment and inexpensive lignin shielding agent (soy protein) will be beneficial to improve the potential accessibility of cellulose in pretreated substrate and reduce the enzyme loading for releasing fermentable sugar from lignocellulose, showing practical significance for promoting the commercialization process of the biofuels industry. 

## Supplementary Materials

The following are available online at https://www.mdpi.com/2073-4360/12/1/191/s1, Table S1: Effects of pretreatment conditions on the content of components in solid substrates, Table S2: Effects of different pretreatments (BA80-W, ET(2) and DA(2) pretreatments) on the total pore volume (cm^3^/g) with pore width below and beyond 5.1 nm, Figure S1: SEM images of (a) bamboo ram material, and substrates pretreated by (b) DA(2), (c) ET(2) and (d) BA80 pretreatments, Figure S2: (a) Cumulative pore volume and (b) incremental pore volume with pore width of bamboo ram material and substrates pretreated by different pretreatments (BA80, ET(2) and DA(2) pretreatments), Figure S3: XPS spectra of bamboo ram material and substrates pretreated by different pretreatments (BA80, ET(2) and DA(2) pretreatments).

## References

[1] Ragauskas A.J., Williams C.K., Davison B.H., Britovsek G., Cairney J., Eckert C.A., Frederick W.J., Hallett J.P., Leak D.J., Liotta C.L. (2006). The path forward for biofuels and biomaterials. Science.

[2] Isikgor F., Becer C.R. (2015). Lignocellulosic biomass: A sustainable platform for the production of bio-based chemicals and polymers. Polym. Chem..

[3] Cherubini F., Jungmeier G., Wellisch M., Willke T., Skiadas I., Van Ree R., de Jong E. (2010). Toward a common classification approach for biorefinery systems. Biofuels Bioprod. Bioref..

[4] Himmel M.E., Ding S.Y., Johnson D.K., Adney W.S., Nimlos M.R., Brady J.W., Foust T.D. (2007). Biomass recalcitrance: Engineering plants and enzymes for biofuels production. Science.

[5] Zhu J.Y., Pan X., Zalesny R.S. (2010). Pretreatment of woody biomass for biofuel production: Energy efficiency, technologies, and recalcitrance. Appl. Microbiol. Biotechnol..

[6] Zhao X.B., Cheng K., Liu D.H. (2009). Organosolv pretreatment of lignocellulosic biomass for enzymatic hydrolysis. Appl. Microbiol. Biotechnol..

[7] Zhao X.B., Li S.M., Wu R.C., Liu D.H. (2017). Organosolv fractionating pretreatment of lignocellulosic biomass for efficient enzymatic saccharification: Chemistry, kinetics, and substrate structures. Biofuels Bioprod. Bioref..

[8] Tang X., Zuo M., Li Z., Liu H., Xiong C., Zeng X., Sun Y., Hu L., Liu S., Lei T. (2017). Green processing of lignocellulosic biomass and its derivatives in deep eutectic solvents. ChemSusChem.

[9] Chen L., Dou J., Ma Q., Li N., Wu R., Yelle D.J., Vuorinen T., Fu S., Pan X., Zhu J.Y. (2017). Rapid and near-complete dissolution of wood lignin at ≤80 °C by a recyclable acid hydrotrope. Sci. Adv..

[10] Hodgdon T., Kaler E. (2007). Hydrotropic solutions. Curr. Opin. Colloid Interface Sci..

[11] Kunz W., Holmberg K., Zemb T. (2016). Hydrotropes. Curr. Opin. Colloid Interface Sci..

[12] Klein-Marcuschamer D., Oleskowicz-Popiel P., Simmons B.A., Blanch H.W. (2012). The challenge of enzyme cost in the production of lignocellulosic biofuels. Biotechnol. Bioeng..

[13] Eriksson T., Börjesson J., Tjerneld F. (2002). Mechanism of surfactant in enzymatic hydrolysis of lignocellulose. Enzyme Microb. Technol..

[14] Yang B., Wyman C.E. (2006). BSA treatment to enhance enzymatic hydrolysis of cellulose in lignin containing substrates. Biotechnol. Bioeng..

[15] Luo X.L., Liu J., Zheng P., Li M., Zhou Y., Huang L., Chen L., Shuai L. (2019). Promoting enzymatic hydrolysis of lignocellulosic biomass by inexpensive soy protein. Biotechnol. Biofuels.

[16] Florencio C., Badino A.C., Farinas C.S. (2016). Soybean protein as a cost-effective lignin-blocking additive for the saccharification of sugarcane bagasse. Bioresour. Technol..

[17] Lin X., Qiu X., Zhu D., Li Z., Zhan N., Zheng J., Lou H., Zhou M., Yang D. (2015). Effect of the molecular structure of lignin-based polyoxyethylene ether on enzymatic hydrolysis efficiency and kinetics of lignocelluloses. Bioresour. Technol..

[18] Cai C., Qiu X., Zeng M., Lin M., Lin X., Lou H., Zhan X., Pang Y., Huang J., Xie L. (2016). Using polyvinylpyrrolidone to enhance the enzymatic hydrolysis of lignocelluloses by reducing the cellulase non-productive adsorption on lignin. Bioresour. Technol..

[19] Akimkulova A., Zhou Y., Zhao X., Liu D. (2016). Improving the enzymatic hydrolysis of dilute acid pretreated wheat straw by metal ion blocking of non-productive cellulase adsorption on lignin. Bioresour. Technol..

[20] Lee W.G., Lee J.S., Lee J.P., Shin C.S., Kim M.S., Park S.C. (1996). Effect of surfactants on ethanol fermentation using glucose and cellulosic hydrolyzates. Biotechnol. Lett..

[21] Sluiter A., Hames B., Ruiz R., Scarlata C., Sluiter J., Templeton D., Crocker D. (2008). Determination of structural carbohydrates and lignin in biomass. Laboratory Analytical Procedure (LAP).

[22] Laine J., Stenius P., Carlsson G., Ström G. (1994). Surface characterization of unbleached kraft pulps by means of ESCA. Cellulose.

[23] Bian H., Chen L., Gleisner R., Dai H., Zhu J.Y. (2017). Producing wood-based nanomaterials by rapid fractionation of wood at 80 °C using a recyclable acid hydrotrop. Green Chem..

[24] Amarasekara A.S., Wiredu B. (2012). A comparison of dilute aqueous *p*-toluenesulfonic and sulfuric acid pretreatments and saccharification of corn stover at moderate temperatures and pressures. Bioresour. Technol..

[25] Grattoni C.A., Dawe R.A., Seah C.Y., Gray J.D. (1993). Lower critical solution coexistence curve and physical properties (density, viscosity, surface tension and interfacial tension) of 2, 6-lutidine plus water. J. Chem. Eng. Data.

[26] Liu J., Hu H., Gong Z., Yang G., Li R., Chen L., Huang L., Luo X.L. (2019). Near-complete removal of non-cellulosic components from bamboo by 1-pentanol induced organosolv pretreatment under mild conditions for robust cellulose enzymatic hydrolysis. Cellulose.

[27] Leschinsky M., Zuckerstätter G., Weber H.K., Patt R., Sixta H. (2008). Effect of autohydrolysis of Eucalyptus globulus wood on lignin structure. Part 2: Influence of autohydrolysis intensity. Holzforschung.

[28] Luo X.L., Liu J., Wang H.S., Huang L.L., Chen L.H. (2014). Comparison of hot-water extraction and steam treatment for production of high purity-grade dissolving pulp from green bamboo. Cellulose.

[29] Donohoe B.S., Decker S.R., Tucker M.P., Himmel M.E., Vinzant T.B. (2008). Visualizing lignin coalescence and migration through maize cell walls following thermochemical pretreatment. Biotechnol. Bioeng..

[30] Gao D., Haarmeyer C., Balan V., Whitehead T.A., Dale B.E., Chundawat S.P. (2014). Lignin triggers irreversible cellulase loss during pretreated lignocellulosic biomass saccharification. Biotechnol. Biofuels.

[31] Yang B., Wyman C.E. (2004). Effect of xylan and lignin removal by batch and flowthrough pretreatment on the enzymatic digestibility of corn stover cellulose. Biotechnol. Bioeng..

[32] Ko J.K., Kim Y., Ximenes E., Ladisch M.R. (2015). Effect of liquid hot water pretreatment severity on properties of hardwood lignin and enzymatic hydrolysis of cellulose. Biotechnol. Bioeng..

[33] Grethlein H.E. (1985). The effect of pore size distribution on the rate of enzymatic hydrolysis of cellulose substrates. Nat. Biotechnol..

[34] Xiang L., Yi Z. (2017). Lignin-enzyme interaction: Mechanism, mitigation approach, modeling, and research prospects. Biotechnol. Adv..

[35] Liu J., Li R.Q., Shuai L., You J.H., Zhao Y.B., Chen L., Li M., Chen L.H., Huang L.L., Luo X.L. (2017). Comparison of liquid hot water (LHW) and high boiling alcohol/water (HBAW) pretreatments for improving enzymatic saccharification of cellulose in bamboo. Ind. Crop. Prod..

[36] Qin C., Clarke K., Li K. (2014). Interactive forces between lignin and cellulase as determined by atomic force microscopy. Biotechnol. Biofuels.

[37] Huang Y., Sun S., Huang C., Yong Q., Elder T., Tu M. (2017). Stimulation and inhibition of enzymatic hydrolysis by organosolv lignins as determined by zeta potential and hydrophobicity. Biotechnol. Biofuels.

[38] Karimi K., Taherzadeh M.J. (2016). A critical review on analysis in pretreatment of lignocelluloses: Degree of polymerization, adsorption/desorption, and accessibility. Bioresour. Technol..

[39] Rollin J.A., Zhu Z., Sathitsuksanoh N., Zhang Y.H. (2015). Increasing cellulose accessibility is more important than removing lignin: A comparison of cellulose solvent-based lignocellulose fractionation and soaking in aqueous ammonia. Biotechnol. Bioeng..

[40] Li M.F., Sun S.N., Xu F., Sun R.C. (2012). Formic acid based organosolv pulping of bamboo (*Phyllostachys acuta*): Comparative characterization of the dissolved lignins with milled wood lignin. Chem. Eng. J..

