# Supplementary material for: Integrating Benzenesulfonic Acid Pretreatment and Bio-Based Lignin-Shielding Agent for Robust Enzymatic Conversion of Cellulose in Bamboo
Source: Polymers (Basel). 2020 Jan 10;12(1):191. doi: 10.3390/polym12010191 (PMC7022729; doi:10.3390/polym12010191)
Supplement: Supplementary file 1 [file polymers-12-00191-s001.pdf]

Supplementary materials

Table S1. Effects of pretreatment conditions on the content of components in solid substrates*.

Sample label[d]	BA content (wt %)	Ethanol loading (g)	H_2SO_4 loading (g)	Pretreatment temperature (°C)	Pretreatment time (min)	Solid substrate yield (%)	The content of components in un- and pretreated substrates (%)[c]		
							Cellulose	Hemicellulose	Klason lignin
RM[a]						100	39.0 ± 0.4[b]	23.8 ± 1.1[b]	26.2 ± 1.0[b]
BA60-T90t30	60			90	30	51.2 ± 0.8	74.8 ± 0.6	12.7 ± 1.7	9.6 ± 1.3
ET(1)-T90t30		23.6	25.1	90	30	66.4 ± 2.2	58.5 ± 1.8	18.1 ± 0.1	21.5 ± 1.4
DA(1)-T90t30			25.1	90	30	85.0 ± 2.1	45.9 ± 1.5	18.7 ± 1.6	28.6 ± 0.9
BA0-T65t30	0			65	30	97.8 ± 3.3	39.8 ± 0.5	23.8 ± 0.3	26.4 ± 0.3
BA10-T65t30	10			65	30	95.6 ± 3.0	40.7 ± 0.4	23.0 ± 0.5	25.3 ± 0.3
BA20-T65t30	20			65	30	92.0 ± 2.5	42.2 ± 0.6	22.7 ± 0.3	23.8 ± 0.9
BA35-T65t30	35			65	30	83.7 ± 2.4	46.2 ± 0.6	21.1 ± 0.5	21.3 ± 0.4
BA50-T65t30	50			65	30	70.0 ± 3.1	55.2 ± 1.7	20.8 ± 0.2	20.3 ± 2.6
BA65-T65t30	65			65	30	62.7 ± 3.5	61.4 ± 1.4	20.1 ± 0.3	15.6 ± 0.9
BA80-T65t30	80			65	30	55.6 ± 3.1	68.7 ± 1.1	19.7 ± 0.6	12.2 ± 1.7
BA90-T65t30	90			65	30	65.0 ± 0.5	59.0 ± 0.6	25.4 ± 1.0	22.7 ± 0.8
BA80-T50t30	80			50	30	76.5 ± 3.9	50.5 ± 1.7	14.6 ± 0.7	23.5 ± 0.8
BA80-T65t30	80			65	30	52.2 ± 2.7	70.4 ± 1.5	13.3 ± 2.0	11.3 ± 1.4
BA80-T80t30	80			80	30	47.6 ± 3.3	79.6 ± 2.6	9.7 ± 1.0	8.1 ± 0.2

BA80-T95t30	80			95	30	42.8 ± 3.5	86.0 ± 4.9	4.9 ± 0.8	5.2 ± 1.1
BA80-T110t30	80			110	30	38.3 ± 3.0	89.8 ± 3.7	3.2 ± 2.5	12.2 ± 1.7
BA80-T50t60	80			50	60	58.3 ± 3.1	65.6 ± 1.8	12.1 ± 0.8	16.4 ± 3.0
BA80-T65t60	80			65	60	50.9 ± 3.3	73.8 ± 3.1	8.3 ± 1.6	10.4 ± 1.1
BA80-T80t60	80			80	60	44.2 ± 2.9	84.5 ± 3.7	4.9 ± 1.3	6.1 ± 1.5
BA80-T95t60	80			95	60	38.2 ± 4.2	91.7 ± 7.4	2.4 ± 1.1	8.4 ± 0.7
BA80-T80t60	80			80	60	43.9 ± 2.4	85.2 ± 3.5	5.0 ± 1.3	5.9 ± 1.8
ET(2)-T80t60		31.5	33.5	80	60	74.6 ± 3.3	51.6 ± 2.0	12.8 ± 1.6	31.2 ± 0.8
DA(2)-T80t60			33.5	80	60	58.5 ± 2.0	65.1 ± 1.9	12.2 ± 1.4	20.6 ± 0.9

[a] RM presents raw material;

[b] on o.d. weight of raw material;

[c] In addition to RM, data for other samples are on o.d. weight of solid substrate.

[d] BA, ET and DA refer to the abbreviations of benzenesulfonic acid, ethanol and dilute acid; the numbers after T and t stand for the pretreatment temperature (°C) and duration (min), respectively.

[*]The ratio of pretreatment liquor to bamboo for all pretreatments are 15 (g:g). The errors appearing in this table represent the standard deviation of the two experimental determinations.

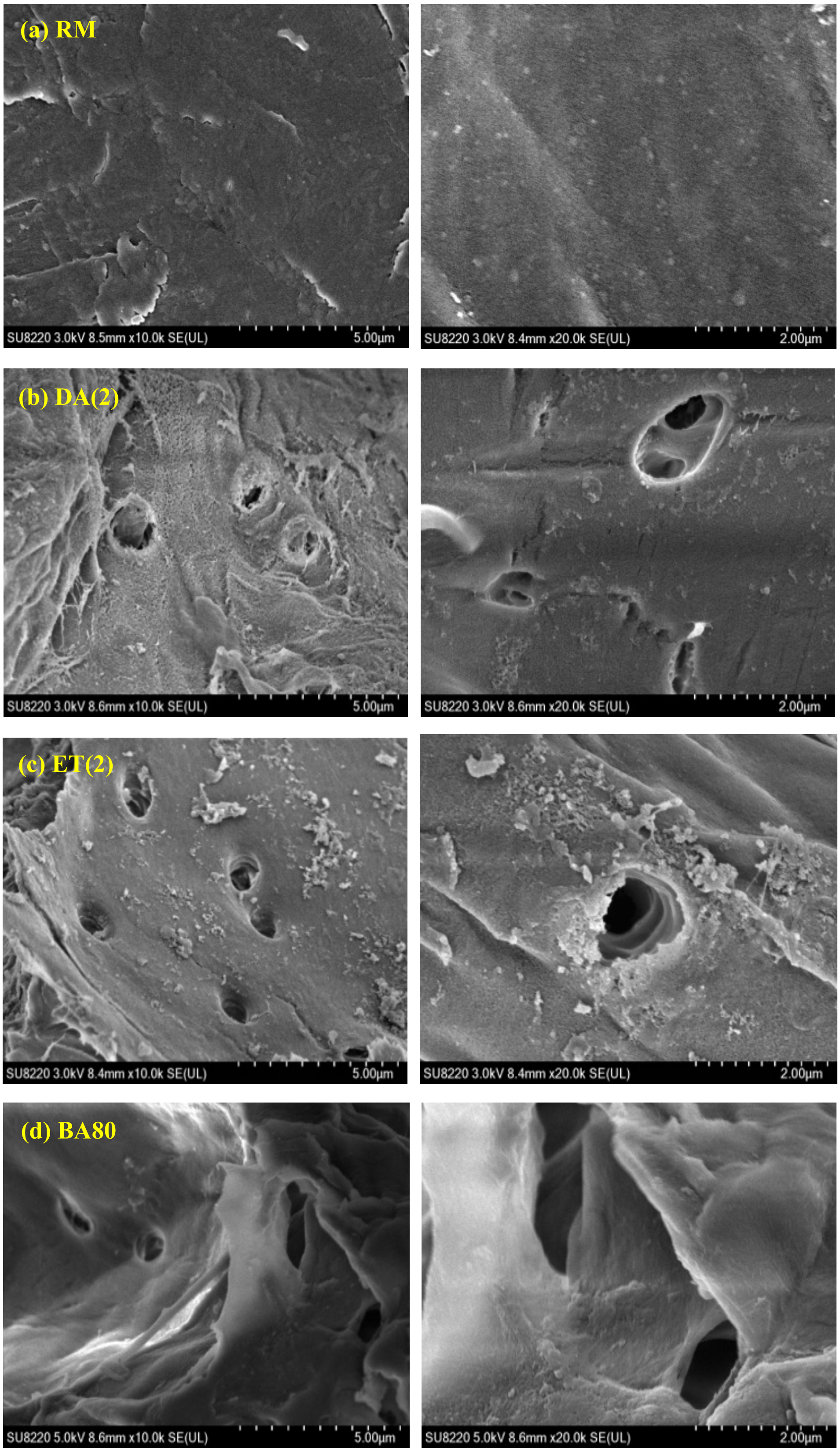

Figure S1. SEM images of (a) bamboo ram material (RM), and substrates pretreated by (b) DA(2), (c) ET(2) and (d) BA80 pretreatments.

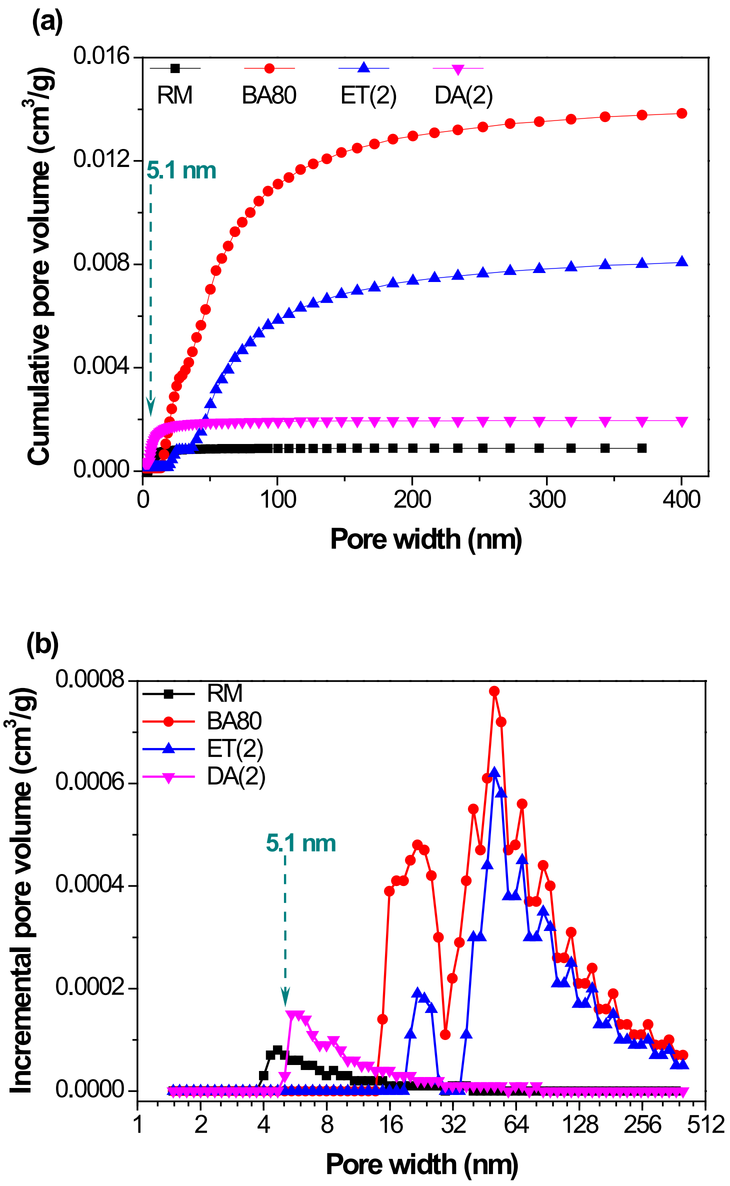

Figure S2. (a) Cumulative pore volume and (b) incremental pore volume with pore width of bamboo raw material (RM) and substrates pretreated by different pretreatments (BA80, ET(2) and DA(2) pretreatments).

Table S2. Effects of different pretreatments (BA80, ET(2) and DA(2) pretreatments) on the total pore volume (cm^3/g) with pore width below and beyond 5.1 nm.

Pore Width (nm)	RM	BA80	ET(2)	DA(2)
< 5.1	0.00025	0.00012	0.00015	0.00037
> 5.1	0.00063	0.01372	0.00792	0.00158

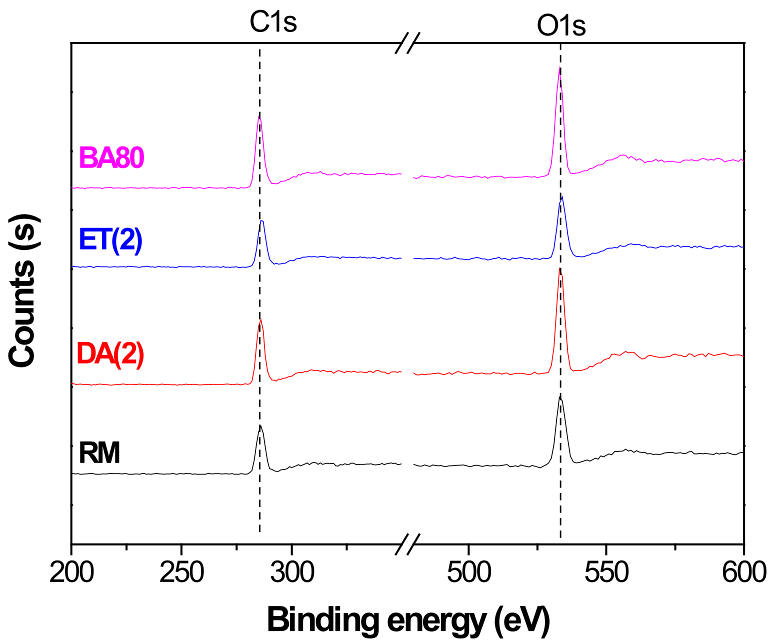

Figure S3. XPS spectra of bamboo ram material (RM) and substrates pretreated by different pretreatments (BA80, ET(2) and DA(2) pretreatments).
